# Different Activity of the Biological Axis VEGF-Flt-1 (*fms*-Like Tyrosine Kinase 1) and CXC Chemokines between Pulmonary Sarcoidosis and Idiopathic Pulmonary Fibrosis: A Bronchoalveolar Lavage Study

**DOI:** 10.1155/2009/537929

**Published:** 2010-02-14

**Authors:** Katerina M. Antoniou, Giannoula Soufla, Athanasia Proklou, George Margaritopoulos, Christiana Choulaki, Rena Lymbouridou, Katerina D. Samara, Demetrios A. Spandidos, Nikolaos M. Siafakas

**Affiliations:** ^1^Department of Thoracic Medicine, University Hospital, Medical School, University of Crete, Heraklion, 71110 Crete, Greece; ^2^Laboratory of Virology, Medical School, University of Crete, Heraklion, Greece; ^3^Department of Rheumatology, Clinical Immunology and Allergy, University Hospital, University of Crete, Heraklion, Greece

## Abstract

*Background*. We have previously shown a different local and systemic angiogenic profile of CXC chemokines in Idiopathic Pulmonary Fibrosis (IPF) patients compared to sarcoidosis. In particular, sarcoidosis showed an angiostatic microenvironment, as compared with the angiogenic cytokine milieu seen in IPF. *Purpose of the Study*. Our aim was to further investigate the aforementioned finding by measuring the expression of different chemokines in granulomatous and fibrotic diseases. We estimated the levels of vascular endothelial growth factor (VEGF) and its high-affinity receptor, Flt-1 (*fms*-like tyrosine kinase 1), in bronchoalveolar lavage fluid (BALF) of patients with IPF and pulmonary sarcoidosis. We have also investigated the mRNA expression of angiogenetic chemokines' receptors such as CXCR2 and CXCR3 and the biological axis of stromal derived factor-1*α* (SDF-1*α* or CXCL12*α*/CXCL12*β*) and receptor, CXCR4. *Methods*. We studied prospectively three groups of patients: (i) one group of 18 patients with IPF, (ii) one group of 16 patients with sarcoidosis, and (iii) 10 normal subjects. *Results*. A statistically significant increase has been detected in VEGF mRNA expression in IPF in comparison with pulmonary sarcoidosis (*P* = .03). In addition, a significant increase has been measured in CXCL12*α* in sarcoidosis in comparison to IPF (*P* = .02). Moreover, a statistically significant decrease has been found in Flt-1 protein levels in pulmonary sarcoidosis in comparison with IPF (*P* = .03). A significant increase in VEGF (*P* = .03) and CXCR4 (*P* = .03) mRNA levels has been also detected in sarcoidosis' patients when compared with healthy controls. *Conclusions*. Our data suggest that increased expression of Flt-1 and downregulation of CXCL12*α* in IPF may further support the hypothesis of a different angiogenetic profile between fibrotic and granulomatous diseases. However, further studies are needed in order to better investigate these enigmatic diseases.

## 1. Introduction

Angiogenesis has been implicated in the pathogenesis of several fibrotic lung conditions, including idiopathic pulmonary fibrosis (IPF). This process plays a significant role in wound healing and contributes to the fibroproliferation and extracellular matrix deposition [1]. Neovascularization in fibroproliferative disorders is regulated by an opposing balance between angiogenic and angiostatic factors [2, 3]. 

Sarcoidosis continues to be a disease of research interest because of its complicated immune mechanisms and elusive etiology [4]. So far, it has been established that granulomatous inflammation in sarcoidosis is predominately a T-helper 1 immune response mediated by a complex network of lymphocytes, macrophages, and cytokines [5]. The cause of progression to a chronic and potentially fibrotic form is still unclear and up to 30% of patients have chronic course of the lung disease, resulting in progressive loss of lung function [6, 7]. 

Recently the chemokine receptors expressed specifically on Th1 cells were identified and it was reported that Th1 cells are characterized by the expression of CCR5 or CXC chemokine receptor 3 [8]. While CXCR3/CXCR3 ligands inhibit angiogenesis, CXCR3 ligands play a pivotal role in orchestrating Th1 cytokine-induced cell-mediated immunity via the recruitment of mononuclear and CD4+ T-cells expressing CXCR3 and consequently via the granuloma formation [4, 5]. So far, there are only few studies in the literature implicating angiogenesis in the immunomodulatory cascade of sarcoidosis. BAL lymphocytes in sarcoidosis have been reported to be highly positive for CCR5 and CXCR3 [9, 10]. Known ligands for CXCR3 are three angiostatic ELR^−^CXC chemokines (Mig/CXCL9, IP-10/CXCL10, and I-TAC/CXCL11) [9–12]. Recent studies have shown that these chemokines play an important role in the accumulation of Th1 lymphocytes in sarcoid lungs [11]. We have shown that sarcoidosis exhibits a distinct angiostatic profile, as shown by an ELR^−^CXC chemokine upregulation in comparison to IPF patients [12]. On the other hand, ELR+ CXC chemokines (IL-8, ENA-78, and GRO-*α*) sharing the same receptor, CXCR2, were found increased in IPF [12], while a downregulation of their levels was recently shown after treatment [13]. 

 Vascular endothelial growth factor (VEGF) is a potent growth factor for endothelial cells that regulates vascular permeability and the stimulation of angiogenesis. VEGF is crucial in lung development and maintenance during the adult life. However, VEGF also contributes in several acute and chronic lung disorders [14]. The biological activity of VEGF depends on its reaction with specific receptors. Two high affinity receptors for VEGF have been described, Flt-1 (*fms*-like tyrosine kinase 1) and KDR/Flk-1 (fetal liver kinase 1) [14, 15]. 

 We hypothesized that the levels of VEGF and receptor Flt-1 would be higher in bronchoalveolar lavage fluid (BALF) from patients with IPF than in patients with pulmonary sarcoidosis. In order to further explore the angiogenetic balance, we measured the mRNA levels of chemokines' receptors, CXCR2 and CXCR3, as well as the biological axis of CXCL12 and cognate receptor, CXCR4.

## 2. Patients

We studied prospectively three groups of patients: (i) 18 patients with IPF, (ii) 16 patients with sarcoidosis, and (iii) 10 normal subjects. Patients' and healthy controls' characteristics are shown in Table 1. 

The diagnosis of IPF was made in 8 cases by surgical biopsy (in the correct clinical context, detailed below) and the histologic diagnosis of Usual Interstitial Pneumonia (UIP) was obtained. In the remaining 10 cases the diagnosis was made on the basis of clinical and high-resolution computed tomography (HRCT) criteria: (1) bilateral basal or widespread crackles, (2) restrictive ventilatory defect or isolated depression of DL_CO_, (3) computed tomography (CT) appearances indicative of IPF with predominantly basal and subpleural microcystic or macrocystic honeycombing, with variably extensive ground-glass and reticular abnormalities but no consolidation, nodular abnormalities, or other parenchymal abnormalities (apart from centrilobular emphysema), and (4) no environmental exposure to a fibrogenic agent or connective tissue disease [16]. According the aforementioned criteria a known cause of pulmonary fibrosis, such as a connective tissue disorder, has been excluded by both immunologic screening and rheumatological clinical evaluation.

Sarcoidosis diagnosis was made according to the ATS/ERS/World Association of Sarcoidosis and Other Granulomatous Disorders joint statement [4]. All patients had transbronchial or open lung biopsy with histopathological evidence of noncaseating epithelioid cell granulomas without evidence of infection or inorganic material to account for the pulmonary granulomatous reaction. According to chest radiographic classification of sarcoidosis, 3 had stage I disease (lymphadenopathy alone), 7 stage II disease (lymphadenopathy and parenchymal opacities), and 6 stage III disease (only parenchymal opacities). 

BALF was also obtained from ten healthy control patients without any past medical history, pulmonary symptoms, or abnormal radiographical findings. 

A former smoker was defined as having smoked at least one cigarette per day for 1 year: smoking histories were collected as part of a routine prospective clinical protocol.

## 3. Methods

### 3.1. Pulmonary Function Tests

All patients were evaluated spirometrically and by measurement of lung volumes, diffusion capacity, and arterial blood gases (at rest). Spirometry and lung volumes (helium-dilution technique) and T_*L*,CO_ (corrected for the haemoglobin) using the single-breath method were performed by a computerised system (Jaeger 2.12; MasterLab, Würzburg, Germany). Predicted values were obtained from the standardised lung function testing of the European Coal and Steel Community, Luxembourg (1993) [17]. Arterial blood gases were measured by an arterial blood gas analyser (AVL330; MasterLab system).

### 3.2. BAL Fluid Processing

BALF was obtained from patients with IPF, sarcoidosis, and from normal healthy controls by methods previously described [12, 13]. Briefly, a flexible bronchoscope was wedged into a subsegmental bronchus of a predetermined region of interest based on radiographical findings. A BAL was performed by instilling a total of 240 mL of normal saline in 60 mL aliquots, each retrieved by low suction. The BALF fractions were pooled and split equally into two samples. One sample was sent to the clinical microbiology and cytology laboratory and the other sample was placed on ice and transported to the research laboratory. The research sample was filtered through sterile gauze (Thompson, Ontario, Canada) and centrifuged at 400 g for 15 minutes at 4°C.

Total cell counts were determined using an improved Neubauer counting chamber and expressed as the total number of cells per mL of aspirated fluid. The pellet was washed three times with cold PBS-Dulbecco's and the cells were adjusted to a final concentration of 10^6^ cells/mL with RPMI1640 plus 2% FCS. The slide preparation was performed as previously reported [18]. The cell-free solution was aliquoted and frozen immediately at -80°C until thawed for chemokine ELISAs.

### 3.3. Assay of Chemokine Levels Using Specific Enzyme-Linked Immunosorbent Assay

Human BALF protein levels of VEGF-A and Flt-1 were quantitated according to the manufacturer's protocol using ELISA kits (R&D Systems) and measurements were performed in the BALF supernatant. Human VEGF-A and Flt-1 had the lowest detectable limit of 5 and 10 pg·mL^−1^, respectively.

### 3.4. RNA Extraction and Reverse Transcription

Total RNA was extracted form each specimen (BALF pellet) using a power homogenizer and the TRIzol reagent (Invitrogen, Carlsband, CA) according to the manufacturer's instructions. cDNA was synthesized using the Strascript reverse transcriptase kit (Stratagene, La Jolla, CA) as previously described [19].

### 3.5. Real-Time RT-PCR

Peptide growth factors mRNA expression was measured using a real-time RT-PCR assay with SYBR-Green I. Primers were designed to span introns [19]. Glyceraldehyde-3-phosphate dehydrogenase (GAPDH) was used as the internal control, in order to normalize VEGF, flt-1, CXCR2, CXCR3, and both transcripts of CXCL12, CXCL12*α* and *β* and CXCR4 expression levels (Table 2). Specifically, 1 *μ*L cDNA from pathological or control samples was amplified in a PCR reaction containing 2X Brilliant SYBR-Green I QPCR Master Mix, 300 nM of each primer and 30 *μ*M ROX passive reference dye, in a final volume of 20 *μ*L. After an initial denaturation at 95°C for 10 minutes, the samples were subjected to 40 cycles of amplification, comprised of denaturation at 95°C for 30 seconds, annealing at appropriate temperature for each primer pair for 30 seconds and elongation at 72°C for 30 seconds, followed by a melt curve analysis, in which the temperature was increased from 55°C to 95°C at a linear rate of 0.2°C /sec. Data collection was performed both during annealing and extension, with two measurements at each step, and at all times during melt curve analysis. In each PCR reaction two nontemplate controls were included. All PCR experiments were conducted on the Mx3000P real-time PCR thermal cycler using the software version 2.00, (Stratagene, La Jolla, CA). To verify the results of the melt curve analysis, PCR products were analyzed by electrophoresis in 2% agarose gels, stained with ethidium bromide, and photographed on a UV light transilluminator. Primer sequences, annealing temperatures, and PCR products length for all the growth factors analyzed, as well as for GAPDH, are described in Table 2.

All reactions were run in triplicates, and peptide growth factor transcript levels were calculated and normalized to each specimen's house keeping gene mRNA (GAPDH) as well as the appropriate calibrators, using the ΔΔCt method for relative quantification. Specifically, after amplification, standard curves were constructed from samples used in a series of consecutive dilutions, for both the gene of interest (GF) and the internal control (GAPDH). Growth factor and GAPDH amplification efficiencies were the same, reaching 100%. IPF and sarcoidosis data were first normalized against variation in sample quality and quantity. Normalized values to GAPDH, ΔCts, were initially calculated using the following equation: ΔCt_sample_ = Ct_GF_ − Ct_GAPDH_.

## 4. Statistical Analysis

Peptide growth factors mRNA levels were first evaluated by the one-sample Kolmogorov-Smirnov goodness of fit test, in order to determine whether they follow a normal or no distribution. Based on the results, the nonparametric Spearman test was used to examine correlations. Proportions were compared using chi-square test. The Kruskal-Wallis followed by a posthoc analysis for pairwise significance and the Mann-Whitney U test were used as indicated to examine growth factors' and chemokines' expression status among IPF and pulmonary sarcoidosis groups. Statistical analysis was carried out using SPSS 13.0 Chicago IL, USA. Statistical significance was set at the 95% level (*P*-value <  .05).

## 5. Results

The demographic and spirometric data of healthy controls, IPF, and sarcoidosis' patients are shown in Table 1. Total cell counts and cell differential were determined and shown in Table 1.

### 5.1. VEGF Expression in mRNA and Protein Level

**Figure 1:**
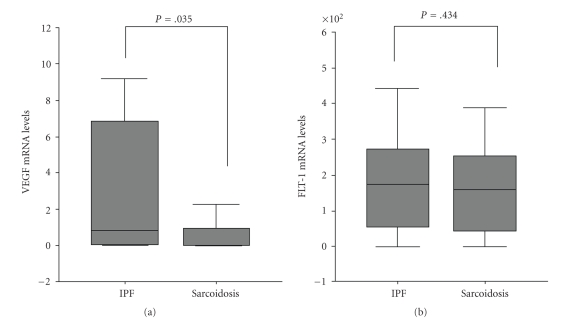
mRNA expression of VEGF/flt-1 in IPF versus Sarcoidosis.

**Figure 2:**
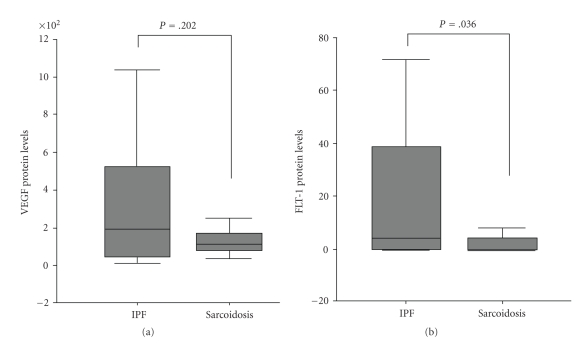
Protein expression of VEGF/Flt-1 in IPF versus Sarcoidosis.

A statistically significant increase has been detected in VEGF mRNA expression in IPF in comparison with pulmonary sarcoidosis (mean ± SD, 18.3 ± 14.9 versus 2.99 ± 1.43, *P* =  .03, resp.) (Table 3, Figure 1). However, no statistically significant difference has been measured in VEGF protein levels between IPF and sarcoidosis patients (mean ± SD, 344 ± 77 versus 154 ± 36, *P* =  .2), suggesting a posttranscriptional decrease of mRNA expression (Table 3, Figure 2). 

A statistically significant increase has been detected in VEGF mRNA expression in sarcoidosis in comparison with healthy subjects (mean ± SD, 2.99 ± 1.43 versus 2.82 ± 3.62, *P* =  .03, resp.). No significant difference has been detected between IPF and control subjects (mean ± SD, 18.3 ± 14.9 versus 2.82 ± 3.62, *P* =  .4, resp.) (Table 4).

### 5.2. Flt-1 Expression in mRNA and Protein Level

No statistically significant difference has been measured in Flt-1 mRNA levels between IPF and sarcoidosis patients (mean ± SD, 154 ± 33 versus 204 ± 52, *P* =  .4) (Table 3, Figure 1). A statistically significant decrease has been detected in Flt-1 protein expression in IPF in comparison with pulmonary sarcoidosis (mean ± SD, 18.8 ± 6.5 versus 3.0 ± 1.4, *P* =  .036, resp.), suggesting a posttranscriptional downregulation of Flt-1 expression (Table 3, Figure 2).

No significant difference has been measured between healthy controls and sarcoidosis or IPF at flt-1 mRNA expression levels (Table 4).

### 5.3. Angiogenetic Chemokines (CXCR2, CXCR3, CXCR4, and CXCL12*α*, CXCL12*β*) mRNA Expression Levels

A significant increase has been measured at CXCL12*α* in sarcoidosis' patients in comparison with IPF samples (Table 4). We have also detected that an increase, however, does not reach statistical significance (*P* =  .06) at mRNA levels of CXCR3 in IPF in comparison with sarcoidosis' patients (Table 4). 

A statistically significant increase has been detected in CXCR4 mRNA expression in sarcoidosis in comparison with healthy subjects (mean ± SD, 2.40 ± 1.02 versus 0.86 ± 0.64, *P* =  .03, resp.). However, no significant difference has been measured between sarcoidosis and healthy controls at mRNA levels of the other chemokines (Table 4).

A significant increase has been detected in healthy controls in comparison with IPF at CXCL12*β* mRNA levels (mean ± SD, 223.8 ± 176.6 versus 141.9 ± 563.2, *P* =  .03, resp.).

## 6. Discussion

To the best of our knowledge this is the first study to investigate the local expression of the biological axis VEGF and its receptor Flt-1 in patients with IPF and Pulmonary Sarcoidosis without pulmonary fibrosis. Our major finding was an increase of the receptor Flt-1 at protein level in IPF in comparison with sarcoidosis. Although the increase in VEGF in IPF patients has not been confirmed at the posttranscriptional analysis, this may be due to high variation in the small group of patients analyzed. In order to further investigate the angiogenetic balance between granulomatous and fibrotic disorders, we measured CXC chemokines' receptors and the biological axis of CXCL12/CXCR4. Our major finding is the significant increase in CXCL12*α* in sarcoidosis' patients in comparison with IPF. 

Recent immunological advances on sarcoidosis have revealed a T helper 1 (Th1) and T helper 2 (Th2) paradigm with predominance of the Th1 response in the immunopathogenesis of sarcoidosis [20, 21]. The concept of disparate activity of the IFN-*γ*-induced CXC chemokines in the context of Th1-like immune disorders, such as sarcoidosis, was originally raised by Agostini et al. who documented an enhanced expression of IP-10 in sarcoid tissues and a positive relationship of BALF IP-10 levels and the degree of T-cell alveolitis, suggesting its pivotal role in ruling the migration of T-cells to sites of ongoing inflammation [22]. Our study group has recently showed a shift versus local Th1 immunologic response in sarcoidosis expressed by upregulation of two major Th1cytokines, IL-12 and IL-18 [23–25]. In addition, we have further supported the assertion that IFN-*γ*-induced CXC chemokines are strongly involved in the immunomodulatory cascade of sarcoidosis implicating angiostasis with Th1 immune response [12]. However, in the current study, the distinct angiostatic profile expressing by CXCR3 between IPF and sarcoidosis did not reach statistical significance. In addition, Miotto et al. [26] described a specific for Th1-mediated response upregulation of IP-10 BALF levels further implicating angiostatic CXC chemokines in the inflammatory cascade of sarcoidosis. Recently, Katoh et al. reported elevated BALF concentrations of IP-10 and MIG in patients with sarcoidosis and chronic eosinophilic pneumonia [27]. Recent data in a large sarcoid population suggest that CXCL9 and CXCL11 are important mediators in recruiting CXCR3-expressing cells [28]. Importantly, it has been shown that both lymphocytes and cells of monocyte lineage express CXCR3 and are involved in the formation of sarcoid lung granulomas [28].

Furthermore, Sekiya et al. [29] demonstrated a strong correlation of elevated VEGF serum levels with clinical parameters of disease activity in sarcoidosis patients indicating a potential usefulness as a predictor of disease activity and responsiveness to treatment. In contrast BAL fluid VEGF levels from sarcoidosis patients were significantly lower than normal controls as reported by Koyama et al. [30]. Low VEGF levels in the lung environment may reduce angiogenesis and induce apoptosis of vascular endothelial cells thus contributing to the pathogenesis of pulmonary sarcoidosis. It has also been suggested that IP-10 may act as a major chemotactic factor for lymphocytes and ENA-78 as a fibrogenic factor, and serum IP-10 levels were more indicative of extrapulmonary lesions [30]. In the current study, we detected an increase of CXCL12*α* and its specific receptor, CXCR4, in pulmonary sarcoidosis, in comparison with IPF and controls, respectively, suggesting its major chemotactic role for lymphocytes, in accordance with the aforementioned recent and previous data [28, 31]. 

However, there are several arguments that should be addressed. First of all, we have the small number of patients included in the study; moreover, there is the limitation of several variations regarding the age, disparity of number of males versus females in each group, smoking status, pulmonary function tests, and BALF cell differentiation between the different disease groups and healthy subjects. Secondly, this study is not a morphological one like other elegant reports in lung tissue [32–35]. On the other hand, this is currently difficult, as the use of lung biopsy for the diagnostic approach of sarcoidosis is limited. In addition, it would be interesting to evaluate the angiogenetic process in different sarcoidosis stages. 

In line with these findings, our study group demonstrated a distinct local angiogenic profile in patients with IPF compared to sarcoidosis patients. The latter evidence implicates angiogenesis in the fibrotic (Th2) pathway of ILDs and highlights novel noninvasive biomarkers to identify patients who are likely to develop progressive disease allowing anti-inflammatory and other treatments to be evaluated or eventually modified before they have failed. However, further studies are needed in order to better investigate these enigmatic diseases.

## Figures and Tables

**Table 1:** Demographic and lung function characteristics of patients with IPF and Pulmonary Sarcoidosis. Values are expressed as mean ± SD, and age as median (range).

Characteristics	Sarcoidosis	IPF	Normal subjects
Number	16	18	10
Sex (Male/Female)	6/10	14/4	5/5
Age median (yrs)	53 (30–64)	69 (56–83)	40 (27–62)
Smokers/ex-	(3/0/13)	(10/2/6)	0/0/10
smokers/Nonsmokers			
FEV_1_ (% pred)	92.1 ± 6.7	82.6 ± 2.6	102 ± 13
FVC (% pred)	94.7 ± 6.2	76.2 ± 2.4	103 ± 19
TLC (% pred)	87.8 ± 3.4	65.4 ± 4.1	95 ± 4
DLco (% pred)	95.1 ± 3.1	52.2 ± 6.1	96 ± 6
PaO_2_ (mmHg)	80.2 ± 5.7	71.5 ± 2.8	85.5 ± 3.1
TCC × 10^5^ /mL	30.7 ± 4.1	24.6 ± 4.1	20.7 ± 5.2
Macrophages%	70.1 ± 8.2	86.1 ± 2.6	94.2 ± 1.3
Neutrophils %	3.2 ± 3.8	6.8 ± 2.1	3.7 ± 2.2
Lymphocytes%	23.8 ± 8.2	5.9 ± 3.8	1.2 ± 1.0
Eosinophils %	1.2 ± 1.9	4.8 ± 2.8	0.3 ± 0.4

TCC: Total cell counts; FVC: Forced Vital Capacity; TLC: Total Lung Capacity; DL_CO_: Diffusing Capacity for Carbon Monoxide; P*α*O_2_: Arterial Partial Pressure of Oxygen.

**Table 2:** Primer sequences used for quantitative Real-time RT-PCR.

Growth factor or Cytokine	Primer pair Sequence (5′-3′)	Annealing temperature	Product size
VEGF	ATGACGAGGGCCTGGAGTGTG	60°C	91
	CCTATGTGCTGGCCTTGGTGAG		
FLT-1	CGGCGGCGGCGAACGAG	58°C	223
	CATGATGTGCTGGGTGCCTTTTA		
CXCL12*α*	TGAGAGCTCGCTTTGAGTGA	55°C	233
	CACCAGGACCTTCTGTGGAT		

CXCL12*β*	CTAGTCAAGTGCGTCCACGA	55°C	221
	GGACACACCACAGCACAAAC		

CXCR2	GGCCACTCCAATAACAGCAGGTC	60°C	197
	GTAGAAAAGGGGGCAGGGTAGAGC		

CXCR3	AAAGCAGAGGGGCAGGCAGCACAC	65°C	181
	AGGGCGGGGAGGTACAGCACGAGT		

CXCR4	GGTGGTCTATGTTGGCGTCT	55°C	229
	TGGAGTGTGACAGCTTGGAG		

**Table 3:** mRNA and protein expression of angiogenetic parameters in IPF and Sarcoidosis. Values are expressed as mean ± SD.

	Sarcoidosis	IPF	*P* value
VEGF-mRNA	2.99 ± 1.43	18.3 ± 14.9	.036
VEGF-protein	154 ± 36	344 ± 77	.202 (NS)
(pg/mL)			
FLT-1-mRNA	204 ± 52	154 ± 33	.434 (NS)
FLT-1protein	3.0 ± 1.4	18.8 ± 6.5	**.036**
(pg/mL)			

NS: Nonsignificant, *P* <.050 is considered statistically significant

**Table 4:** mRNA expression of VEGF, FLT-1 and angiogenetic chemokines in healthy subjects (Controls), Sarcoidosis (SARC) and IPF patients. Values are expressed as mean ± SD.

Variables	Controls	IPF	SARC	*P*1 value	*P*2 value	*P*3 value
VEGF	2.82 ± 3.6	18.3 ± 14.9	2.99 ± 1.43	NS	.03	.03
Flt-1	134.6 ± 23.5	154.1 ± 33.5	204 ± 52	NS	NS	NS
CXCR2	0.001 ± 0.002	0.08 ± 0.25	0.0004 ± 0.001	NS	NS	NS
CXCR3	0.03 ± 0.002	0.12 ± 0.39	0.0001 ± 0.0004	NS	NS	NS
CXCR4	0.86 ± 0.64	174.3 ± 543.6	153.9 ± 129.3	NS	.03	NS
CXCL12*α*	92.2 ± 132.17	168.5 ± 576.7	1525.2 ± 2224.7	NS	NS	.02
CXCL12*β*	223.8 ± 176.6	142.4 ± 563.7	822.4 ± 1423.0	NS	NS	NS

NS: Non significant, *P* < .050 is considered statistically significant,

*P*1: *P* value between controls and IPF,

*P*2: *P* value between controls and Sarcoidosis,

*P*3: *P* value between IPF and Sarcoidosis.

## Abbreviations

ACE: Angiotensin Converting Enzyme
BALF: Bronchoalveolar Lavage Fluid
CXCL: CXC ligand
CXCR: CXC receptor
ELISA: Enzyme-linked immunosorbent assay
FEV_1_: Forced Expiratory Volume in 1 second
FVC: Forced Vital Capacity
Kco: Carbon monoxide transfer coefficient
IFN-*γ*: Interferon-gamma
IPF: Idiopathic Pulmonary Fibrosis
Th1: T helper 1
VEGF: Vascular Endothelial Growth Factor
UIP: Usual Interstitial Pneumonia
EAA: Extrinsic Allergic Alveolitis
fIIPS: fibrotic Idiopathic Interstitial Pneumonias
fNSIP: fibrotic Nonspecific Interstitial Pneumonia
FLT-1: *fms*-like tyrosine kinase 1
RT PCR: real time
GAPDH: Glyceraldehyde-3-phosphate dehydrogenase
ATS: American Thoracic Society
ERS: European Respiratory Society
KDR/Flk-1: fetal liver kinase 1
COP: Cryptogenic Organizing Pneumonia
ILDs: Interstitial Lung Diseases.
